# Correlating Coating Characteristics with the Performance of Drug-Coated Balloons – A Comparative *In Vitro* Investigation of Own Established Hydrogel- and Ionic Liquid-Based Coating Matrices

**DOI:** 10.1371/journal.pone.0116080

**Published:** 2015-03-03

**Authors:** Sebastian Kaule, Ingo Minrath, Florian Stein, Udo Kragl, Wolfram Schmidt, Klaus-Peter Schmitz, Katrin Sternberg, Svea Petersen

**Affiliations:** 1 Institute for Biomedical Engineering, University of Rostock, Friedrich-Barnewitz-Straße 4, 18119, Rostock, Germany; 2 Institute for Chemistry—Analytical and Technical Chemistry, University of Rostock, Albert-Einstein-Straße 3a, 18059, Rostock, Germany; University of California, Merced, UNITED STATES

## Abstract

Drug-coated balloons (DCB), which have emerged as a therapeutic alternative to drug-eluting stents in percutaneous cardiovascular intervention, are well described with regard to clinical efficacy and safety within a number of clinical studies. *In vitro* studies elucidating the correlation between coating additive and DCB performance are however rare but considered important for the understanding of DCB requirements and the improvement of established DCB. In this regard, we examined three different DCB-systems, which were developed in former studies based on the ionic liquid cetylpyridinium salicylate, the body-own hydrogel hyaluronic acid and the pharmaceutically well-established hydrogel polyvinylpyrrolidone, considering coating morphology, coating thickness, drug-loss, drug-transfer to the vessel wall, residual drug-concentration on the balloon surface and entire drug-load during simulated use in an *in vitro* vessel model. Moreover, we investigated particle release of the different DCB during simulated use and determined the influence of the three coatings on the mechanical behavior of the balloon catheter. We could show that coating characteristics can be indeed correlated with the performance of DCB. For instance, paclitaxel incorporation in the matrix can reduce the drug wash-off and benefit a high drug transfer. Additionally, a thin coating with a smooth surface and high but delayed solubility can reduce drug wash-off and decrease particle burden. As a result, we suggest that it is very important to characterize DCB in terms of mentioned properties *in vitro* in addition to their clinical efficacy in order to better understand their function and provide more data for the clinicians to improve the tool of DCB in coronary angioplasty.

## Introduction

In recent years, drug-coated balloons (DCB) providing a short-term transfer of the antiproliferative drug paclitaxel (PTX) to the arterial wall have emerged as an upcoming therapeutic alternative to drug-eluting stents (DES) in the field of vascular intervention [1]. In fact, they shall reduce the ongoing complications of in-stent restenosis and late stent thrombosis associated with stent implantation by a high initial drug delivery to the vessel wall with little impact on long-term healing [2]. Furthermore, the absence of a remaining foreign body in the artery and high deliverability are opening the opportunity of their use in small vessels, bifurcations or long lesions, which are hardly accessible by stents [2] [3]. In the past ten years a lot of clinical research has been done to prove the clinical efficacy of DCB either in comparison to bare metal stents (BMS) or DES [4] [5]. However, the development of DCB is complex as especially the coating should be robust enough to physically maintain the drug on the surface of the balloon during transit of the device through hemostatic valves, introducers, guiding catheters and the vascular system while still allowing a rapid, uniform and efficient drug transfer to the vessel wall during balloon dilatation. As aforementioned the currently engaged drug is PTX, which has proven a rapid uptake by the intima, a high retention rate and a sustained biological effect [6]. Often, transfer agents are additionally applied in order to enhance the drug transfer capability [1] [7]. Some of them are well studied as e.g. the contrast agent iopromide [8], urea [9] and plasticizers [10].

While DES coatings are designed as a drug reservoir in order to allow a sustained drug release over a long time period [11], described coating matrices used for DCB are rather hydrophilic and loose to guarantee an entire drug transfer during balloon dilatation. For that reason especially drug wash-off rates during transit of the device through the vascular system should not be underestimated. For instance, while Kelsch *et al*. [5] reported PTX wash-off rates of up to 26% and 36% for urea- or iopromide-based DCB, respectively, Berg *et al*. [7] observed drug wash-off rates of 42% for an iopromide-based formulation during an *in vitro* passage through a hemostatic valve and a guiding catheter.

In this regard, we developed and established three different matrices for DCB based on (i) the ionic liquid (IL) cetylpyridinium salicylate (Cetpyrsal) [12], (ii) the natural and body-own hydrogel hyaluronic acid (HA) [13] and (iii) the synthetic but pharmaceutically often applied hydrogel polyvinylpyrrolidone (PVP) [14] in previous studies. We considered IL as attractive matrix for DCB, because important physical, chemical and biological properties are tunable [15] by combination of various e.g. pharmaceutical active anions and cations within a broad range [16] [17]. The use of a body-own hence highly biocompatible coating like HA or pharmaceutically well-established PVP as drug reservoir and transfer agent was assumed of high interest for DCB, having in mind that many of the interventional cardiovascular devices already incorporate a hydrophilic lubricious coating in order to ease movement through the vasculature [18] [19].

In our first *in vitro* studies, the IL- and both hydrogel-based coatings evidenced good performance with regard to drug loss and transfer. For example, we determined drug wash-off rates of 28% for the Cetpyrsal/PTX, < 5% for the HA/PTX and 34% for the PVP/PTX coating and drug transfer rates of 40%, 50% and 49%, respectively [12] [13] [14]. Although values are not comparable as different *in vitro* models were used for their determination, we like to conclude from these data that the application of either IL or hydrogels approved as promising matrices for DCB at first sight. Chosen additives seem to indeed guarantee good adherence of the drug to the balloon surface by low water solubility and efficient drug transfer upon balloon dilatation by swelling combined with drug elution upon compression.

Within this study, we compare the three established DCB with the aim of correlating coating characteristics with DCB performance, which should be considered as highly important for the understanding of the DCB coating requirements and hence the improvement of established DCB. In this regard, we provide identical conventional balloon catheter with the different coatings and evaluate their coating morphology, coating thickness, drug loss, drug transfer, and residual drug load as well as particle release during simulated use in the same *in vitro* vessel model. Moreover, we determine the influence of the three coating additives on the mechanical behavior of the balloon catheter. Besides the aforementioned correlation of DCB properties with their performance, this thorough *in vitro* evaluation, considering the specific requirements and challenges of DCB, should furthermore provide a testing platform for further DCB.

## Experimentals

### Materials

All chemicals were purchased from Sigma-Aldrich (Taufkirchen, Germany), Mallinckrodt Baker (Griesheim, Germany), SERVA Feinbiochemica (Heidelberg, Germany), Thermo Scientific (Karlsruhe, Germany), or Merck (Darmstadt, Germany) in p.a. quality or higher if not indicated differently.

HA sodium salt from *streptococcus equi* (Mw = 1,500,000 g/mol, <1% protein impurities, Fluka, Taufkirchen, Germany), PVP (K90, Mw = 360,000 g/mol, Sigma-Aldrich, Taufkirchen, Germany), Cetpyrsal (self-synthesized, see following paragraph) and PTX (>99.5%, Cfm Oskar Tropitzsch e.K., Marktredwitz, Germany) were used as coating matrices and model drug, respectively.

For the characterization of the coating thickness, tubes of polyetherblockamide (PEBAX 7033 SA01), known as standard material for the balloons of balloon catheters, of 5 mm in length and diameter (A_o_ = 78.5 mm²), provided by Biotronik SE & Co. KG (Erlangen, Germany), were drawn on stainless steel bars of the same diameter (5 mm) for better handling and used as model balloon surface.

For all other experiments folded and uncoated PEBAX balloon catheters of 4 mm in diameter (nominal diameter in expanded state) and 30 mm in length (A_o_ = 377.0 mm²) were kindly provided by Biotronik SE & Co. KG.

### Synthesis of Cetpyrsal

Cetpyrsal was synthesized according to Bica et al. [20] and Petersen *et. al*. [12]. Briefly, 10.32 g cetylpyridinium chloride and 4.48 g of sodium salicylate were dissolved in 50 mL of water/acetone (1:1, v/v) and the solution was stirred at 23°C ± 2°C overnight. Subsequently, 100 mL of water was added to the reaction solution prior to extraction with dichloromethane for at least 5 times. In order to remove residual sodium chloride (NaCl), the combined extracts were washed with water until no more chloride ions could be detected in the washings (checked by addition of AgNO_3_ solution). Finally, the extract was dried over a molecular sieve and dichloromethane was evaporated under reduced pressure. The resulting Cetpyrsal was a crystalline slightly yellow powder. ^1^H and ^13^C NMR spectra were recorded in DMSO-d6 at 23°C on a Bruker AVANCE 300 III spectrometer (Coventry, UK) for verification of the structure.

### Preparation of different PTX-loaded coatings on tubular PEBAX and on balloon catheter

#### Cetpyrsal-based coating

Firstly, PTX and Cetpyrsal were dissolved separately in methanol (MeOH) to obtain concentrations of 4.72 mg/mL. Secondly, the PTX solution was diluted 1:1 with the Cetpyrsal solution in order to obtain a PTX concentration of 50% (w) in Cetpyrsal. Thirdly, we slowly pipetted 100 μL of the resulting Cetpyrsal-PTX solution on the PEBAX tube (78.5 mm^2^) in order to acquire the established PTX surface load of 3 μg/mm^2^ [21]. The volume of 100 μL turned out to be best manageable for the coating of a surface of the aforementioned 78.5 mm^2^ in preliminary experiments [12]. The constant PTX concentration of 2.36 mg/mL in the solution thus resulted from the predefined volume of 100 μL and PTX surface load of 3 μg/mm^2^. For the coating of balloon catheters, which were left in folded condition, the pipetted volume was adapted in accordance to the surface to be covered. During the manual pipetting the tubes as well as the balloons were rotated in a weak air stream to guarantee a full evaporation of the solvent. Finally, all coatings were dried at 23°C ± 2°C overnight.

#### HA-based coating

The HA-based coating was developed and optimized with regard to adherence to the balloon surface and crosslinking condition in our previous research [13]. Optimized coating conditions were applied here. Briefly, we silanized the PEBAX tubes, drawn on stainless steel bars, or the balloon catheter with 3-glycidoxypropyl-trimethoxysilane (GPTMS) to enhance the adhesion of the HA coating on the substrate. Therefore, PEBAX samples were treated by O_2_-plasma at 45 W for 3 min with a radio frequency plasma generator (frequency 13.56 MHz, Diener electronic GmbH & Co. KG, Ebhausen, Germany) at a low pressure of 0.3 mbar in order to generate free hydroxyl groups on the surface. For subsequent silanization, 4 mL of 1% (w) GPTMS dissolved in dry toluene was poured over each sample and stirred for 16 h at 23°C ± 2°C. Finally, the samples were rinsed with water and dried in a vacuum chamber at 40°C and 50 mbar overnight.

As a second step, we attached a HA base layer to the silanized PEBAX surfaces. This chemical attachment was achieved via direct reaction of terminal epoxy groups at the modified surface and hydroxyl groups of HA and afforded no crosslinker. Therefore, tubes or balloons were immersed into 1 mL or respectively 4 mL of distilled water (dH_2_O) containing 5 mg/mL HA after adjustment to pH 6 by adding 0.1 M HCl. After 1 h reaction at 65°C, samples were washed three times with Dulbecco’s phosphate buffered saline (DPBS, pH 7.2) containing 0.05% (w) polyoxyethylene (20) sorbitan monolaurate (Tween 20) and dH_2_O and dried in a vacuum chamber at 40°C and 50 mbar overnight.

After chemical attachment of the HA base layer, further HA layers were deposited to the modified surfaces via manual dip coating. After each dipping of the tube or the balloon into either 1 mL or 4 mL of 5 mg/mL HA in DPBS for 30 s, samples were immersed for 5 min into a 1 mL or respectively 4 mL 1 mM N-(3-dimethylaminopropyl)-N’-ethylcarbodiimide hydrochloride (EDC) and N-hydroxysuccinimide (NHS) solution in DPBS for crosslinking. The dipping process was repeated ten times with drying for 5 min at 23°C ± 2°C after each dipping step. Afterwards, samples were washed again three times with DPBS/Tween 20 and dH_2_O and dried in a vacuum chamber at 40°C and 50 mbar overnight.

In contrast to our previous study, PTX incorporation was performed via pipetting after the drying process in order to guarantee a consistent surface load of 3 μg/mm^2^. Therefore, PTX was dissolved in ethanol/dH_2_O (8/2 (v/v)) mixture to yield a concentration of 2.36 mg/mL. 100 μL of this solution were then slowly pipetted per PEBAX tube under rotation in a weak air stream. The solvent mixture has been chosen as it provides good PTX stability and enough swelling of the hydrogel allowing interpenetration of the drug [14]. For the coating of balloon catheters, which were left in folded condition, the pipetted volume was adapted in accordance to the surface to be covered. Finally, all coatings were again dried in a vacuum chamber at 23°C ± 2°C overnight.

#### PVP-based coating

For improved PVP covering of PEBAX tubes or balloon catheter, we established a spray-coating process in previous researches [14]. Briefly, PVP was dissolved in chloroform to yield a concentration of 0.25% (w). For the spraying, either the PEBAX tubes, drawn on stainless steel bars, or the balloon catheter were inserted into a holder of an electro pneumatic airbrush system, which guaranteed homogeneous coating by continuous rotation along their longitudinal axis. By means of coating-intermediate weighing using a microbalance (UMX5, Mettler Toledo, Giessen, Germany), we determined the spraying time needed for the deposition of total coating mass of 706 μg for the PEBAX tubes and 3390 μg for the balloon catheter. Prior to crosslinking, all coatings were dried in a vacuum chamber at 40°C and 50 mbar overnight.

Crosslinking of the PVP coatings was performed by irradiations using a 3UV lamp (Ultra Violet Products, P/N 95-0343-02, 254-302-365 nm, 8 W/230 V, ∼50 Hz/0.16 A, Cambridge, UK) after drying and prior to PTX incorporation via pipetting. Therefore, coated PEBAX tubes or balloon catheters were placed underneath the lamp in a distance of 15 mm. The lamp of 8 W was set to 254 nm, which produces a radiant flux of 1670 μW/cm² at 2’’ distance (manufacturer specification). The PEBAX tubes or the balloon catheter were rotated intermediately during the 10 min crosslinking process. Higher wavelengths and shorter irradiation times resulted in inefficient crosslinking, as evidenced in former studies [14].

PTX incorporation was performed via pipetting after the drying process in order to guarantee a consistent surface load of 3 μg/mm^2^ in accordance to [14] and the described PTX incorporation within the HA coating.

### Characterization

#### Scanning electron microscopy

Coating morphologies were examined in a Philips XL 30 Environmental Scanning Electron Microscope (Philips Electron Optics, Eindhoven, The Netherlands) operating in the ESEM mode with a water vapor pressure of 1.2 mbar. The accelerating voltage was fixed to 10 kV and the beam current to 11 μA. The working distance was adapted to each sample and varied from 9.5 mm to 23.7 mm, as indicated as WD in the legend of each micrograph. Samples were attached to the specimen mount as obtained after the coating process and examined at four different positions along the coating.

#### Confocal microscopy

**Fig 1:**
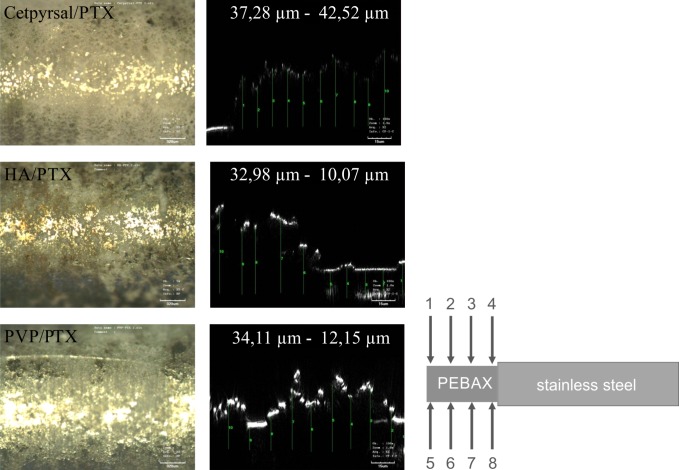
Coating thickness of differently coated DCB. Representative light microscopic micrographs (left) and height profiles (xz scans, right) determined by confocal microscopy of Cetpyrsal/PTX coating (top), HA/PTX coating (middle) and PVP/PTX coating (bottom). The indicated thickness ± SD has been averaged over 8 positions along the PEBAX tube fitted to a stainless steel pin with ten measurement points each (right).

Coating thickness of the different PTX-loaded coatings was evaluated with PEBAX-tubes pulled on stainless steel bars for better handling in the confocal microscope. The coating thickness was measured at eight positions along the PEBAX surface (Fig. 1) by means of the microscope LEXT OLS 3000 (Olympus, Hamburg, Germany). We determined the coating thickness at all eight positions by ten different measurements applying the lens Plan Achromat MP lan Apo 100x numerical aperture 0.95. Since drug-loaded coatings were opaque and thus not penetrable with the laser light it was necessary to generate small defects on the homogeneous coatings to measure the coating thickness at the one and the other side of the defect.

### Simulated use of different DCB in an in vitro vessel model

#### Drug loss, transfer and residual load

We simulated the process of a coronary balloon angioplasty using an *in vitro* model which has been recently described in literature [22]. The model consists of a guiding catheter (Cordis Vista Brite Tip; 5 F; 100 cm; ID = 1.4 mm) with a guide wire (Biotronik Galeo M014) and a tortuous path revetted with a PTFE tube. The anatomic model was equivalent to the model described in ASTM F2394-07, X.2.4 surrounded by 37°C ± 2°C heated water. The connection between the guiding catheter and the inner PTFE tubing of the vessel model was sealed to avoid fluid loss or contamination of the inner lumen of the test path. On the distal end of the test path a silicone test tube (ID = 3 mm) was placed as model of the target vessel. All test samples were manually advanced over the guide wire through the guiding catheter and the test path of the model until the balloon was placed in the silicone tube. The guiding catheter and vessel model were then flushed with 30 mL of 0.9% NaCl to recover particles and PTX lost during tracking (“after track 1”). Afterwards the balloon was dilated to its nominal pressure of 7 bar and held for 30 s. After deflation of the balloon, the solution contained in the silicone tube was collected and filled up to 30 mL for particle and drug analysis (“after dilatation 1”), while the silicone tube was removed from the vessel model and stored in an empty flask. The balloon was cut and also stored in an empty flask for morphology assessment and residual drug content determination. The entire test path was then flushed with 30 mL MeOH for determination of the residual PTX content lost during tracking (“after track 2”) and afterwards with 0.9% NaCl in preparation of the next test.

The cut balloon and the silicone tube were then extracted for 30 min with 20 mL of MeOH at 23°C ± 2°C (“residual balloon drug load”, “after dilatation 2”). Then the drug content in all collected solutions (“after track 1”, “after track 2”, “after dilatation 1”, “after dilatation 2” and “residual balloon drug load”) was determined by means of HPLC after 1:10 dilution with MeOH using conditions described in the following section. The determined drug content in the collected solutions “after track 1” and “after track 2” as well as in the solutions “after dilatation 1” and “after dilatation 2” are summed up and presented in the results as drug loss during track and drug transfer in silicone tube, respectively. The calculated total drug load is the sum of all measured PTX amounts. We evaluated the drug loss, transfer and load of the different DCB with n = 5 balloons per coating.

#### HPLC parameters

20 μL of the test solutions were injected into an Eurospher column 100-5, C18, 120 × 4 mm ID (Wissenschaftlicher Gerätebau Dr.-Ing. Herbert Knauer GmbH, Berlin, Germany). The chromatographic conditions were: column temperature 23°C, isocratic eluent PBS (0.005 M, pH 3.5)—acetonitrile 50–50% (v/v), flow rate 1.0 mL/min and UV detection at 230 nm with calibrated measurement range 0.5–20.0 mg/L and detection limit approximately 0.05 mg/L.

*Particle counting test*: Sub visible particles (≥10 μm and ≥25 μm) were analyzed according to USP 788 „particulate matter in injections” and were adopted from assessment of surface and coating damage of stent delivery catheters using the particle counter HIAC ROYCO 9703 device (sensor model HRLD400, HACH, Loveland, Colorado, USA). The vials to be measured were filled with 30 mL of test liquid (“after track 1”, “after dilatation 1”). Before measurement all vials were degassed for 1 h at 23°C ± 2°C and carefully inverted 20 times to provide homogenous distribution of particles in the test solution. Visual investigation of the whole solution was made to check for visible particulate matter or other irregularities. Measurement of sub visible particles was performed on 4 test portions with 5 mL of the whole test liquid each. The result from the first test portion was discarded and only measurements 2–4 were used for averaging in order to ensure equilibration of the measurement system prior to particle analysis. The results were calculated for the total volume of 30 mL, thus representing the total burden received from the balloon. We evaluated the particle loss and transfer of the different DCB with n = 5 balloons per coating.

#### Investigation of the mechanical performance of different DCB

We characterized the influence of the different PTX-loaded coatings on the mechanical behavior of the balloon catheter by measuring two different functional parameters recently described in literature [22]: the trackability and crossability of the catheters. For the measurements, each DCB was introduced into a guiding catheter (Cordis Vista Brite Tip; 5 F; 100 cm; ID = 1.4 mm) over a 0.014’’ guide wire (Biotronik Galeo M014) to complete the interventional system. Neither the guiding catheter nor the guide wire was changed during testing to ensure a constant test environment. All measurements were performed in a 37°C heated water basin at a travel speed of 7.5 mm/s delivered by a guide (maximum travel distance 500 mm, Cleveland Präzisionssysteme GmbH, Löffingen, Germany), which is driven by a DC servo motor (type tendo PM 41, Chr. Mayr GmbH, Mauerstetten, Germany).

The details of both investigated mechanical properties are given in the following. The guiding catheter and the vessel model were flushed prior to each testing series with 50 mL dH_2_O. Mechanical tests were estimated using vessel models developed by Schmidt et al., the models were derived from typical vessel anatomy but adapted to the individual tests [23].

#### Measurement of Trackability

According to Schmidt *et. al*. [23], we considered the trackability as the ability of the DCB to track or move easily through a curved vascular pathway. To characterize the trackability we used the same set up as Schmidt *et. al*. In fact, the proximal push force required to advance the DCB through the tubing system was measured. The trackability indicates the resistance force at the proximal end of the DCB moving through a curved vessel pathway. The lower the push force, the better is the trackability [22]. All measurements of forces were performed using a standardized experimental model of the left coronary circulation developed in the Institute of ImplantTechnology und Biomaterials e.V. (IIB).

**Fig 2:**
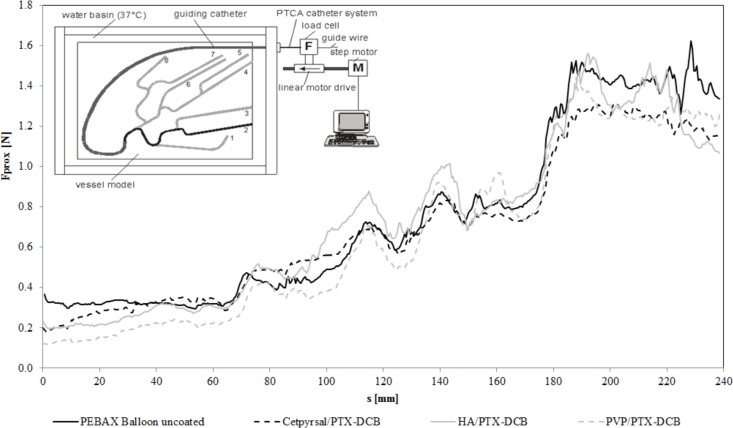
Trackability of differently coated DCB. Trackability as the resulting force at the proximal end of the catheter during the passage of a vessel model, diagram shows force-distance curves of differently coated balloon catheter in comparison to an uncoated catheter of the same type. The insert shows a scheme of the test set up according to [23]. In fact, the curves show averages of 3 experiments per balloon type (3 different coatings or uncoated).

The model consists of two PMMA plates (thickness: 10 mm), with a milled path for the guiding catheter, which leads to the model of the coronary arteries. The two PMMA plates are pinned and bolted together. The diameter of the path for the guiding catheter is 2 mm, and the milled path diameter for the coronary arteries is 2.5 mm (Fig. 2). Out of the total of eight vessel paths, we have chosen test path 2 for the characterization of the trackability of the DCB. Previous experiments with stent delivery systems have shown that this is the most challenging path to pass [22]. The investigated DCB were advanced over the test path from point A, which is still inside of the guiding catheter, until the tip of the balloon catheter has reached point E. The total travel distance was 240 mm. All measurements were performed in a 37°C heated water environment with n = 3 balloons of each DCB variant. To obtain a measure of the DCB’s trackability, the proximal track forces F_prox_ of each single measurement were averaged from all three measurements, and a mean track force was calculated from the curves as follows:

$$\mathrm{Trackability}:\sum_{i=1}^{n}F_{p\text{rox}}(i)\text{where n is the number of force measurements along the entire travel path}\;\left(\text{n}=440\right).$$

#### Measurement of Crossability

**Fig 3:**
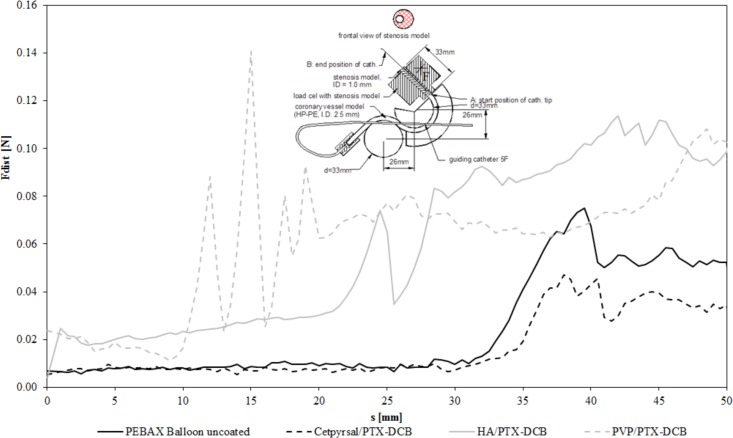
Crossability of differently coated DCB. Crossability as the resulting force at the distal end of the catheter during the passage of a model stenosis, diagram shows force-distance curves of differently coated balloon catheter in comparison to an uncoated catheter of the same type. The insert shows a scheme of the test set up according to [23]. In fact, the curves show averages of 3 experiments per balloon type (3 different coatings or uncoated).

Also according to Schmidt *et. al*. [23], we considered the crossability as the ability of DCB to pass through the target lesion. To characterize the crossability we used again the same experimental set up as Schmidt e. al. [22]. In fact the distal reactive force developed during the crossing maneuver was measured. The lower the reactive force developed during crossing of the lesion, the better is the crossability [22]. To measure the crossability, the experimental setup used during investigation of the trackability was modified by integrating a stenotic lesion model at the end of the test path (Fig. 3). This lesion model from PMMA is characterized by an eccentric narrowing of the vessel lumen from 2.5 mm to 1.0 mm with a conical transition. A load cell was mounted to the lesion model to provide measurements of the distal reactive force F_dist_ in addition to the proximal push force F_prox_. The proximal travel distance (driven by the linear actuator) was 50 mm beginning at the end of the vessel simulation (point A) until the entire balloon has passed the stenosis model (point B). All measurements were performed in a 37°C heated water environment with n = 3 balloons of each DCB variant. The distal reactive forces were averaged over the three measurements. To obtain a measure of the DCB’s crossability, the distal cross forces F_dist_ of each single measurement were averaged from all three measurements, and a mean cross force was calculated from the curves as follows:

$$\mathrm{Crossability}:\sum_{i=1}^{n}F_{\mathrm{dist}}(i)$$

where n is the number of force measurements along the entire travel path (n = 50).

#### Statistics

Mean values and standard deviations (SD) were analyzed using IBM SPSS software 20.0. Indicated statistical differences were analyzed using the non-directional Mann–Whitney U-test, assuming p<0.05 as significantly different and p<0.01 as very significantly different.

## Results

### Surface morphology of different DCB

**Fig 4:**
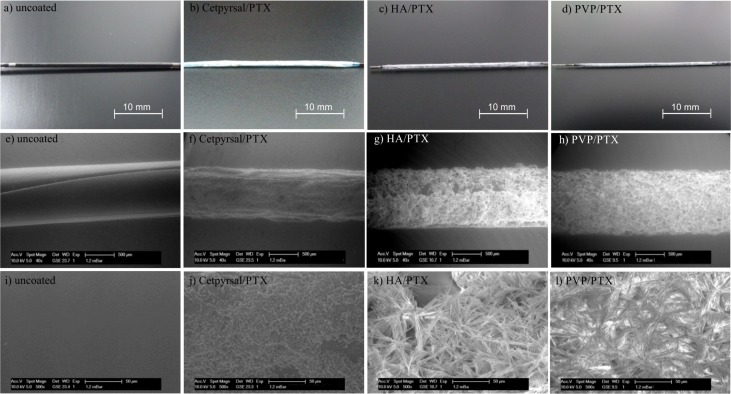
Morphological analysis of differently coated DCB. Representative photos (a-d) and ESEM-micrographs (e-l) for the examination of the surface morphology of differently coated balloon catheter in comparison to an uncoated balloon, e-h: magnification 40x, i-l: magnification 500x.

We assessed the surface morphology of differently coated DCB in comparison to an uncoated balloon via electron microscopy. Representative ESEM micrographs (Fig. 4) revealed a homogeneous coating for the three compared DCB. In fact, the coating integrity appears complete and homogenously distributed irrespectively of the coating matrix. The morphology of the Cetpyrsal/PTX DCB is uniform, no differences between the drug and the matrix are recognizable and no PTX crystals are visible. In contrast, the HA/PTX coating shows a different morphology. Large PTX crystals, which are only attached to the balloon surface and not incorporated within the HA matrix, dominate the micrograph. In comparison, the PVP/PTX coating depicted a similar morphology. The PTX also formed large crystalline structures but in contrast to HA the PVP is more likely incorporating the drug.

### Coating thickness of different DCB

We evaluated the coating thickness of the three mentioned DCB by means of confocal height profiles with ten measurements at eight positions along the PEBAX tube. Fig. 1 shows representative height profiles. The coating thickness of the Cetpyrsal/PTX coating is lower and seems more uniform in contrast to the two hydrogel coatings, visualized by the observed lower standard deviation. In fact, we measured a coating thickness of 18.94 μm ± 3.40 μm for the Cetpyrsal/PTX coating, 32.98 μm ± 10.07 μm for the HA/PTX coating and 34.11 μm ± 12.15 μm for the PVP/PTX coating.

### In vitro PTX loss and transfer of different DCB during simulated use

**Fig 5:**
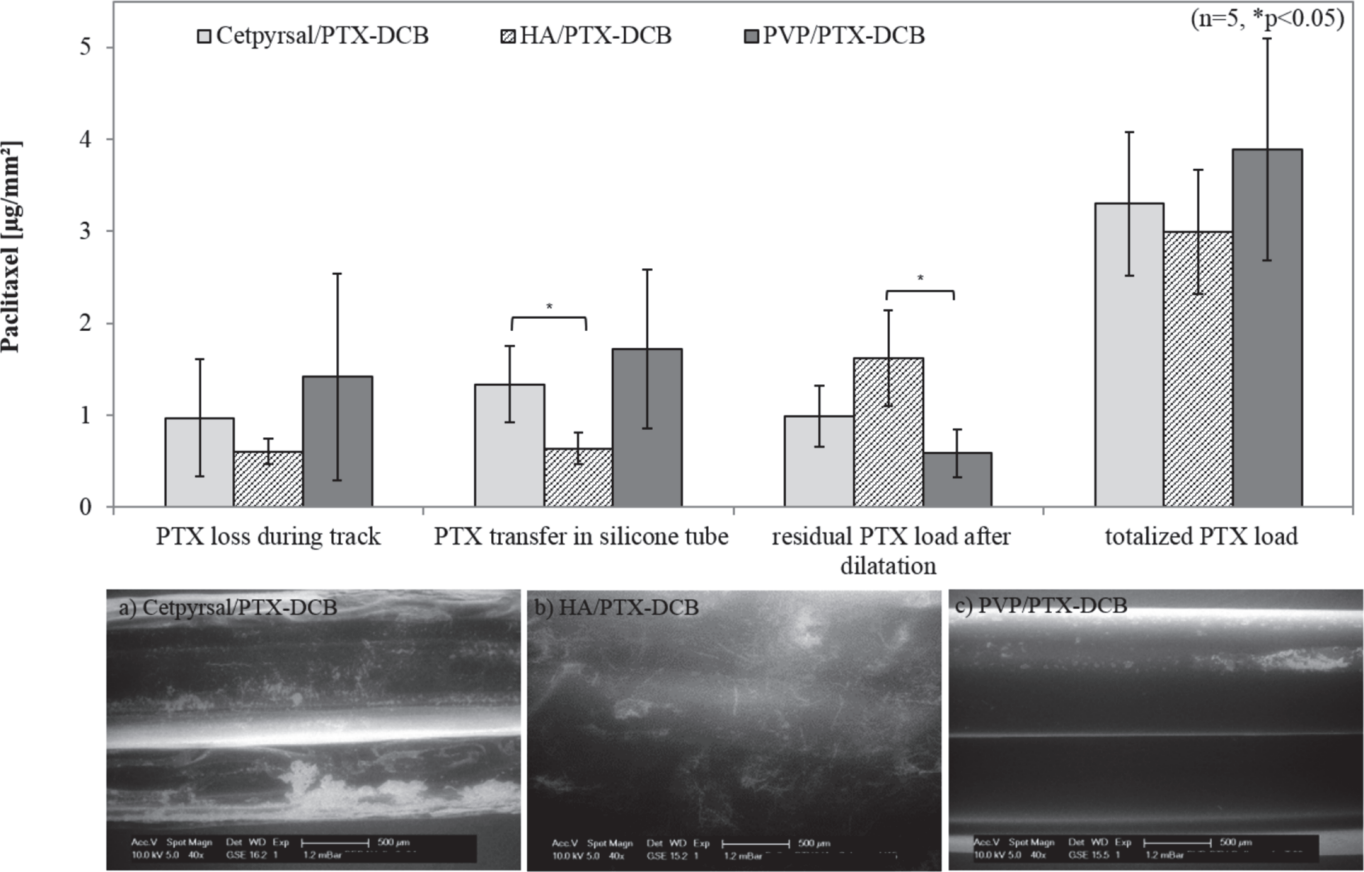
Results of simulated use test of differently coated DCB. Drug loss during track, drug transfer in silicone tube, residual drug load and totalized drug load of differently coated DCB after simulated use in a vessel model according to ASTM F2394-07, X.2.4. Bars show mean ± SD of 5 experiments (* p<0.05), a-c: representative ESEM-micrographs of the differently coated balloon catheter after simulated use in the vessel model.

In order to simulate the use of the different DCB in a vessel model, we advanced the balloon catheter manually through a vessel model, consisting of a guiding catheter with a guide wire and a tortuous vessel path under measurement of drug loss and transfer during transit of the balloon. This *in vitro* test set up should include the mechanical stress on the coating during insertion of the catheter to the area of clinical interest. We observed a drug wash off of 28% for the Cetpyrsal/PTX DCB, 21% for the HA/PTX DCB and 39% for the PVP/PTX DCB (Fig. 5). Furthermore, the Cetpyrsal/PTX DCB could deposit 40% of its entire drug load in the exemplary vessel wall (silicone tube) during dilatation, while we quantified a drug transfer of 22% for the HA/PTX DCB and 44% for the PVP/PTX DCB. After dilatation we determined the residual drug load on the balloon in order to characterize the efficacy of the different systems. In conclusion, we ascertained residual drug loads of 32% on Cetpyrsal/PTX DCB, 54% on HA/PTX DCB and 17% on PVP/PTX DCB.

After passage through the vessel model and dilatation the different DCB show a uniform coating appearance without any cracks, see Fig. 5A-C. In comparison to the detected residual drug loads, the ESEM micrographs underline the measurements. The Cetpyrsal/PTX coating shows some brighter, nearly white, areas which are probably generated from the remaining PTX on the balloon surface. Similarly the HA/PTX coating shows also some brighter areas. In addition, some residual PTX crystals can be seen on the surface what again underlines the highest residual drug load measured in this test. The PVP/PTX coating only shows a few brighter areas on the top side of the balloon what corroborates the lowest residual drug load of 17% measured in this test.

### In vitro particle loss and transfer of different DCB during simulated use

Additionally to the drug loss and transfer, we measured the loss and transfer of particles (≥ 10 μm, ≥ 25 μm) during track and upon balloon expansion. As aforementioned these size limits are adopted from assessment of surface and coating damage of stent delivery catheters [24]. The results for the particle counting test are given in Table 1 and given in number of particles per mm² balloon surface (#/mm²). The Cetpyrsal/PTX coating lost 100 ± 106 particles **≥** 10 μm and 16 ± 12 particles **≥** 25 μm. In contrast, the HA/PTX coating lost 418 ± 198 particles **≥** 10 μm and 25 ± 9 particles **≥** 25 μm while the PVP/PTX coating lost 790 ± 191 particles **≥** 10 μm and 53 ± 11 particles **≥** 25 μm. Furthermore, we detected 70 ± 37 particles **≥** 10 μm and 18 ± 10 particles **≥** 25 μm transferred from the Cetpyrsal/PTX DCB during dilatation, 37 ± 7 particles **≥** 10 μm and 3 ± 1 particles **≥** 25 μm transferred from the HA/PTX DCB and 93 ± 53 particles **≥** 10 μm and 12 ± 7 particles **≥** 25 μm transferred from the PVP/PTX DCB, respectively.

**Table 1:** Particles lost and released after simulated use in in vitro vessel model.

		Results			Statistical Analysis		
	Particle Size [μm]	Cetpyrsal/PTX-DCB	HA/PTX-DCB	PVP/PTX-DCB	Cetpyrsal/PTX- vs. HA/PTX-DCB	Cetpyrsal/PTX-vs. PVP/PTX-DCB	HA/PTX- vs. PVP/PTX-DCB
Number of particles lost during track [#/mm²]	≥10	100 ± 106	418 ± 198	790 ± 191	**	**	*
	≥25	16 ± 12	25 ± 9	53 ± 11	n.s.	**	**
Number of particles transferred after dilatation [#/mm²]	≥10	70 ± 37	37 ± 7	93 ± 53	n.s.	n.s.	n.s.
	≥25	18 ± 10	3 ± 1	12 ± 7	**	n.s.	**

Number of particles (≥10 μm, ≥25 μm) per mm² balloon surface released from differently coated DCB after simulated use in an *in vitro* vessel model according to ASTM F2394-07, X.2.4. Values show mean ± SD of 5 experiments

n.s.: not significant

* significant: p<0.05

** very significant: p<0.01

### Trackability of different DCB

The resulting force–distance graphs were derived from the passage of the different DCB through the test path 2 of the vessel model described in [22], see Fig. 2. The initially low track forces began to rise as the distance of DCB advancement increased. Only negligible differences were observed between the differently coated DCB and in comparison to the uncoated balloon catheter of the same type. The mean track forces ranged from 0.69 N ± 0.36 N for the Cetpyrsal/PTX DCB over 0.73 N ± 0.41 N for the HA/PTX DCB to 0.66 N ± 0.43 N for the PVP/PTX DCB. For comparison, a mean track force of 0.75 N ± 0.43 N was determined for the uncoated reference balloon catheter.

### Crossability of different DCB

The measured distal reactive forces F_dist_ of the DCB and the uncoated balloon catheter across the total distance of 50 mm (A to B) required to pass through the stenotic lesion (ID = 1 mm) are given in Fig. 3. In contrast to the Trackability, the mean crossing forces for the DCB ranged from 0.02 N ± 0.01 N for the Cetpyrsal/PTX DCB over 0.07 N ± 0.04 N for the HA/PTX DCB to 0.06 N ± 0.03 N for the PVP/PTX DCB. For comparison, a mean crossing force of 0.02 N ± 0.02 N was determined for the uncoated reference balloon catheter.

## Discussion

In order to correlate DCB coating characteristics with performance regarding drug delivery, which should be considered as highly important for the understanding of the DCB coating requirements and hence the improvement of established DCB, we provide a thorough *in vitro* evaluation at the example of three different DCB matrices. Because we established our own coatings we were able to guarantee optimized coating conditions. In addition, using the same type of balloon catheter for every experiment allows us to compare the three different matrices objectively and independently of the catheter characteristics.

The first coating characteristic investigated, was the coating morphology evaluated by electron microscopy. Here, the Cetpyrsal/PTX coating seems to differ from both hydrogel coatings. While Cetpyrsal and PTX form a nearly homogenous coating matrix, PTX crystals are clearly distinguishable from both smooth hydrogels (Fig. 4). We assume that this difference results from the one or two step coating technique. While the Cetpyrsal/PTX coating is pipetted in one step from a single solution, the hydrogel-based DCB were coated in a two-step process firstly either dipping (HA) or spraying (PVP) and secondly pipetting the PTX on the hydrogel coating. The two step process was however necessary when applying hydrogels in order to provide sufficient crosslinking and thereby decreased water solubility of hydrogels afforded for minimized drug loss during simulated use as shown in previous studies [13] [14] without affecting PTX availability and stability. For instance, applying PTX with HA or PVP in one step and performing hydrogel crosslinking with EDC/NHS or UV light irradiation, respectively, in presence of PTX might either cause its crosslinking within the hydrogels as described in [13] [14] [25] [26] or in case of UV light irradiation even its photodecomposition [14] [27] [28]. Both processes would lower the bioavailability of PTX and thereby possibly DCB efficacy.

Considering differences between the two hydrogel coatings, we determined a different grade of incorporation of the PTX crystals into the matrix. Although both coatings show nearly the same thickness (Fig. 1) and the PTX solution pipetted as second step was the same, the PVP seems to better surround the PTX crystals than HA. Searching for an explanation, we measured the coating thickness without PTX to underline the statement of different grades of drug incorporation into the two hydrogels. As mentioned in [13], the coating thickness of the HA coating without PTX is about 2.8 μm. In contrast, we measured a coating thickness of about 9.7 μm for the PVP coating without PTX incorporation (S1 Table) which is nearly a triple of the coating thickness and may additionally underline that PVP can surround the drug better than HA. Furthermore, we compared the different swelling properties of HA and PVP from previous and other own experiments. In fact, a water uptake of 5.0 mg per mg initial coating mass of the HA coating [13] and 7.5 mg per mg initial coating mass of the PVP coating [14] after 1 min elution in DPBS was detected, which is equal to the time needed for the coating of one balloon. Although swelling of the two hydrogels in EtOH/H_2_O is estimated to be considerable lower, the same tendency may be expected, giving indication for better drug incorporation within PVP.

The second characteristic investigated was coating thickness after drug incorporation. The observed differences in drug integration from the morphological analyses are also recognizable in coating thickness measurements. The better integration of PTX into Cetpyrsal results in a decreased coating thickness (Cetpyrsal/PTX: 18.9 μm ± 3.4 μm, HA/PTX: 33.0 μm ± 10.1 μm, 34.1 μm ± 12.2 μm) as well as a decreased standard deviation in contrast to the two hydrogels.

For DCB performance, we detected a correlation of drug loss and transfer with coating morphology. Obviously a good incorporation of PTX within the matrix, as seen in Fig. 4 for the Cetpyrsal/PTX and PVP/PTX coatings, refers to a high drug transfer during dilatation, as results of 40% for Cetpyrsal/PTX DCB and 44% for PVP/PTX DCB evidence. Drug transfer of Cetpyrsal/PTX DCB is even significantly higher (p = 0.016) in comparison to HA/PTX DCB (22%) where the PTX crystals are not incorporated properly into the matrix.

However, also drug loss enhanced from 21% for HA/PTX DCB, to 28% for Cetpyrsal/PTX DCB to 39% for PVP/PTX DCB, see Fig. 5, probably due to an increased water uptake which benefits both drug loss as well as drug transfer. If PTX is not well incorporated into the matrix, it builds a kind of lipophilic barrier and inhibits the water uptake as shown for HA/PTX DCB. Furthermore, the nearly pure PTX sticks on the HA coated balloon surface and cannot be transferred to the vessel wall during dilatation. Additionally, a residual drug load of 54% for the HA/PTX DCB (32% for Cetpyrsal/PTX DCB, 17% for PVP/PTX DCB, Fig. 5) underlines this hypothesis.

Besides incorporation, drug loss seems to be furthermore defined and influenced by other coating characteristics. For instance, increasing drug wash-off rates are assumed to be associated with rough surfaces and enhanced coating thicknesses as a result of increasing friction when the balloon is passing the access path and the arterial system. Accordingly, we observed a lower drug loss of 28% for the smooth and thin Cetpyrsal/PTX coating (18.94 μm ± 3.40 μm) compared to 39% determined for the rough and thicker PVP/PTX coating (34.11 μm ± 12.15 μm), see Fig. 1,4 and 5.

The overall objectives when developing a DCB are the combination of a low drug loss and a high drug transfer. Results of our study verify that this is best achievable via good PTX incorporation within the coating matrix, low surface roughness and thin coating. In this context, among tested specimens especially the Cetpyrsal/PTX DCB but also the PVP/PTX DCB show promising drug delivery characteristics in comparison to established DCB. During simulated use in the *in vitro* vessel model, we determined PTX losses of 28% and 39% of the entire drug load which are in the same range as above mentioned PTX losses of 26%–42% reported in literature for balloons coated with urea or iopromide, respectively, during *in vitro* passage through a hemostatic valve and a guiding catheter [5] [7]. However, mechanical strain on the coating in our study can be estimated higher due to the additional passage of the DCB through the tortuous path simulating the anatomy of coronary arteries. Regarding drug transfer of established DCB to the vessel wall *in vivo*, 20% of initial PTX load 15–20 min and 17% of initial PTX load 40–60 min post stent implantation were reported by Kelsch *et. al*. [5] and Scheller *et. al*. [8], respectively. Own results of 40% drug transfer for Cetpyrsal/PTX DCB and 44% for PVP/PTX DCB in a silicone tube, which are similar to data obtained in the previous *in vitro* studies [12] [14], are hence promising, although data might again not be directly correlated to *in vivo* performance.

The HA/PTX DCB evidence lower drug transfer rates what does not directly correlate with former published data in [13] where we determined a promising drug loss < 5% and transfer rates of about 50%. The difference might be a result of the testing method. In our former studies we did not simulate the use of the DCB in a tracking model. Drug wash-off was only determined by simple elution for 1 min in DPBS and drug transfer via subsequent dilation in a silicone tube. In fact, mechanical strain, which has an enormous influence on the wash-off rate, was not considered. Furthermore, the wash-off due to elution was probably that low because of the lipophilic barrier built from non-incorporated PTX crystals. Consequently, the drug transfer during subsequent dilatation and cracking of the coating benefitted from the low wash-off in this experimental set-up. Hence we assume that the reported high drug transfer rate in the former study can be rather compared to the sum of both parameters, drug loss and drug transfer observed in the actual set-up involving the simulation of the insertion procedure within the *in vitro* vessel model.

As further performance parameter of DCB, we measured the generation of particles ≥10 μm and ≥25 μm during transit of the balloon and upon balloon dilatation in the stenotic model. While the estimated mechanism of drug delivery from DCB indeed involves delivery of drug particles to the inner lumen of coronary arteries, release of drug particles or coating fragments in the coronary arteries might be associated with complications such as occlusions of small vessels or capillaries, possibly leading to micro embolization [21]. For the Cetpyrsal/PTX coating it can be assumed that all measured particles are most likely PTX particles since we could determine that Cetpyrsal does not generate any detectable particles in aqueous medium at 38 μg/mL, corresponding to the maximal obtainable concentration during our set-up [12]. This is in accordance with the obtained result that the summed amount of particles lost and released upon dilatation from Cetpyrsal/PTX DCB is the lowest among tested DCB (Cetpyrsal/PTX: 204 ± 165 #/mm², HA/PTX: 483 ± 215 #/mm², PVP/PTX: 1124 ± 83 #/mm²), see Table 1. The number of lost and released particles from hydrogel-based coatings is very significantly higher (p = 0.008) which does not correlate with determined drug loss and drug release results. It could hence be assumed that measured particles are also generated from the coating matrices. Undoubtedly, crosslinking of the hydrogels either through EDC/NHC (HA) or UV (PVP) led to an insolubility of the polymers in aqueous media. Besides the effects of coating matrix, the morphology of the DCB coating has an influence on the lost and released particles. Especially when PTX forms large crystals, it can be expected that the number of lost and released particles exceeding diameters of 10 μm increases as shown for Cetpyrsal/PTX DCB with a total amount of 204 ± 165 particles #/mm² in comparison to PVP/PTX DCB with a total amount of 1124 ± 83 #/mm² particles.

Interestingly, the particle burden of Cetpyrsal/PTX DCB is even lower in comparison to commercially available DCB with an urea-based coating (340 #/mm², data not shown), tested under same experimental conditions, and corresponding in vivo studies concerning efficacy and safety of the same commercial DCB are favorable [2], PTX particle burden of the developed Cetpyrsal-based DCB is not expected to be harmful.

In addition to the drug delivery performance, we considered the influence of the different drug-loaded matrices on the mechanical behavior of the balloon catheter as an important factor. If the balloon catheter is only hardly deliverable through the vascular system, mechanical stress and friction would increase which could lead to a severe injury of the vessel lumen. Moreover, the balloon coating might be harmed resulting in increased drug wash-off rates which on the other hand would decrease the drug transfer and furthermore the efficacy of the DCB.

During the transit of the tortuous path, we observed a mentionable difference in trackability of the different DCB or in comparison to an uncoated balloon catheter of the same type. The force-distance curves of the trackability measurements reflect the geometry of the tortuous test path. The increasing forces at 80 mm, 115 mm and 140 mm travel distance represent the passage of the balloon at the respective curves of the vessel model (Fig. 2).

In comparison, differences between the different DCB and the uncoated reference balloon catheter when passaging the stenotic model during the performed crossability test could be determined. We assume that because the coating thickness of the hydrogel-coated DCB, thus the outer diameter of the balloon, is more than doubled, the difference in distal reaction force while passing the lesion is up to 0.04 N higher for the hydrogel-coated DCB (Fig. 3). In addition, a rougher surface morphology of the hydrogel-coated DCB might enhance the friction of the coated balloon in the target vessel. But as seen from the results, the differences were probably inconsiderable during further *in vivo* experiments. Obviously, the PVP/PTX DCB had minor problems entering the stenotic model although we used a conventional guide wire resulting in force peaks between 10 mm and 20 mm.

To summarize we conclude that the studied coating matrices had only a minor influence on the mechanical behavior of the balloon catheter because the coated area respectively the coated balloon is very small in contrast to the balloon shaft and the catheter. As a result we could not detect decreased frictional properties of the hydrogel-coated balloons, as one might have imagined, as the influence of the coated balloon on the catheter is negligible.

## Conclusion

Within this study, we evaluated three new coating matrices developed in former studies concerning their coating morphology and coating thickness and investigated their performance during a simulated angioplasty in an *in vitro* vessel model according to ASTM standards. Additionally, we examined the influence of the coatings on the mechanic behavior of the balloon catheter. In particular, we have shown that different additives lead to DCB with different coating morphologies and thicknesses, drug wash-off rates, drug transfer rates and particle burden. As a result, we worked out the following requirements for good DCB performance, being low drug-wash and high drug transfer during dilatation combined with low particle burden: (i) good PTX incorporation within the coating, (ii) thin coating with a smooth surface and (iii) good but delayed water solubility of the coating additive. This kind of solubility is important for the transfer of the drug into the stenosis, on the one hand the coating should not dissolve during tracking or before dilatation but on the other hand a water insoluble coating could generate particles due to friction within the vasculature. Among tested matrices, the IL Cetpyrsal combines all these requirements and thus achieved efficient drug transfer at low particle burden. For sure, further applicability of all presented coating designs concerning sterilizability and storage stability but most notably the *in vivo* safety and efficacy of the developed DCB however affords further research.

To summarize, it is very important to characterize DCB in terms of mentioned properties in addition to their clinical efficacy clarifying the differences between a well and insufficient performing DCB system, e.g. higher drug loss during transit of the balloon than during dilatation. We hereby suggest a series of methods to evaluate either established DCB as well as new developed ones concerning an efficient use, to work out differences between especially the matrices and to provide more data for the clinicians to improve the tool of DCB in coronary angioplasty.

## Supporting Information

S1 Table Coating Thickness of PVP-Coated PEBAX-Tubes. Coating Thickness of PVP-Coated PEBAX-Tubes without PTX incorporation, the indicated thickness ± SD has been averaged over 8 positions along the PEBAX tube fitted to a stainless steel pin with ten measurement points each.
(PDF)
