# Supplementary material for: Correlating Coating Characteristics with the Performance of Drug-Coated Balloons – A Comparative In Vitro Investigation of Own Established Hydrogel- and Ionic Liquid-Based Coating Matrices
Source: PLoS One. 2015 Mar 3;10(3):e0116080. doi: 10.1371/journal.pone.0116080 (PMC4348426; doi:10.1371/journal.pone.0116080)
Supplement: S1 Table — Coating Thickness of PVP-Coated PEBAX-Tubes without PTX incorporation, the indicated thickness ± SD has been averaged over 8 positions along the PEBAX tube fitted to a stainless steel pin with ten measurement points each. (PDF) [file pone.0116080.s001.pdf]

Coating Thickness of PVP-Coated PEBAX-Tubes

9.7	±	1.9	µm

Sample 1 11.1 ± 1.3 µm

MP1

Result	Off	Off
Upper limit		
Standard		
Lower limit		
#	10.0	10
Average	11.1	1.5
Stdabw	1.3	0
Min.	9.2	1.5
Range	4.2	0
Sigma	1.3	0
3 Sigma	3.9	0

#	Judge	Thick[um]	RefractiveIndex
1		10.9	1.5
2		9.6	1.5
3		12.5	1.5
4		9.2	1.5
5		11.1	1.5
6		10.1	1.5
7		10.8	1.5
8		11.8	1.5
9		13.4	1.5
10		11.3	1.5

MP2

Result	Off	Off
Upper limit		
Standard		
Lower limit		
#	10.0	10
Average	11.6	1.5
Stdabw	0.5	0
Min.	10.8	1.5
Range	1.5	0
Sigma	0.5	0
3 Sigma	1.6	0

#	Judge	Thick[um]	RefractiveIndex
1		11.5	1.5
2		10.8	1.5
3		10.8	1.5
4		11.5	1.5
5		12.3	1.5
6		11.7	1.5
7		11.7	1.5
8		11.3	1.5
9		12.1	1.5
10		12.4	1.5

MP3

Result	Off	Off
Upper limit		
Standard		
Lower limit		
#	10	10
Average	11.9	1.5
Stdabw	0.5	0
Min.	10.8	1.5
Range	1.8	0
Sigma	0.5	0
3 Sigma	1.4	0

#	Judge	Thick[um]	RefractiveIndex
1		10.8	1.5
2		11.9	1.5
3		12.0	1.5
4		12.0	1.5
5		11.8	1.5
6		11.7	1.5
7		12.1	1.5
8		12.7	1.5
9		11.8	1.5
10		12.4	1.5

MP4

Result	Off	Off
Upper limit		
Standard		
Lower limit		
#	10	10
Average	11.9	1.5
Stdabw	0.6	0
Min.	10.8	1.5
Range	2.1	0
Sigma	0.6	0
3 Sigma	1.8	0

#	Judge	Thick[um]	RefractiveIndex
1		11.6	1.5
2		12.9	1.5
3		12.1	1.5
4		11.9	1.5
5		12.0	1.5
6		11.0	1.5
7		12.1	1.5
8		12.4	1.5
9		12.0	1.5
10		10.8	1.5

MP5

Result	Off	Off
Upper limit		
Standard		
Lower limit		
#	10	10
Average	11.1	1.5
Stdabw	1.0	0
Min.	9.9	1.5
Range	2.9	0
Sigma	1.0	0
3 Sigma	3.0	0

#	Judge	Thick[um]	RefractiveIndex
1		10.4	1.5
2		10.2	1.5
3		11.7	1.5
4		10.1	1.5
5		12.8	1.5
6		10.9	1.5
7		9.9	1.5
8		11.3	1.5
9		11.9	1.5
10		12.2	1.5

MP6

Result	Off	Off
Upper limit		
Standard		
Lower limit		
#	10	10
Average	10.8	1.5
Stdabw	1.0	0
Min.	9.1	1.5
Range	3.0	0
Sigma	1.0	0
3 Sigma	3.0	0

#	Judge	Thick[um]	RefractiveIndex
1		10.7	1.5
2		10.7	1.5
3		9.5	1.5
4		10.4	1.5
5		11.7	1.5
6		12.1	1.5
7		10.9	1.5
8		12.0	1.5
9		10.9	1.5
10		9.1	1.5

MP7

Result	Off	Off
Upper limit		
Standard		
Lower limit		
#	10	10
Average	10.2	1.5
Stdabw	0.8	0
Min.	9.2	1.5
Range	2.6	0
Sigma	0.8	0
3 Sigma	2.3	0

#	Judge	Thick[um]	RefractiveIndex
1		11.8	1.5
2		10.3	1.5
3		9.4	1.5
4		9.8	1.5
5		9.8	1.5
6		10.4	1.5
7		10.7	1.5
8		9.2	1.5
9		10.6	1.5
10		9.7	1.5

MP8

Result	Off	Off
Upper limit		
Standard		
Lower limit		
#	10	10
Average	10.6	1.5
Stdabw	2.4	0
Min.	6.7	1.5
Range	6.9	0
Sigma	2.4	0
3 Sigma	7.3	0

#	Judge	Thick[um]	RefractiveIndex
1		6.7	1.5
2		8.2	1.5
3		7.4	1.5
4		11.2	1.5
5		12.2	1.5
6		10.0	1.5
7		12.2	1.5
8		11.7	1.5
9		13.1	1.5
10		13.6	1.5

Sample 2 8.2 ± 1.1 µm

MP1

Result	Off	Off
Upper limit		
Standard		
Lower limit		
#	10	10
Average	8.6	1.5
Stdabw	0.8	0
Min.	7.2	1.5
Range	2.6	0
Sigma	0.8	0
3 Sigma	2.5	0

#	Judge	Thick[um]	RefractiveIndex
1		7.8	1.5
2		7.2	1.5
3		9.8	1.5
4		8.0	1.5
5		9.1	1.5
6		9.2	1.5
7		8.0	1.5
8		8.5	1.5
9		9.5	1.5
10		8.6	1.5

MP2

Result	Off	Off
Upper limit		
Standard		
Lower limit		
#	10	10
Average	7.8	1.5
Stdabw	1.4	
Min.	5.9	1.5
Range	5.0	0
Sigma	1.4	0
3 Sigma	4.2	0

#	Judge	Thick[um]	RefractiveIndex
1		5.9	1.5
2		8.7	1.5
3		10.9	1.5
4		6.7	1.5
5		7.1	1.5
6		7.0	1.5
7		8.6	1.5
8		7.4	1.5
9		8.0	1.5
10		7.5	1.5

MP3

Result	Off	Off
Upper limit		
Standard		
Lower limit		
#	10	10
Average	9.1	1.5
Stdabw	0.7	0
Min.	8.0	1.5
Range	2.2	0
Sigma	0.7	0
3 Sigma	2.1	0

#	Judge	Thick[um]	RefractiveIndex
1		9.9	1.5
2		8.6	1.5
3		8.0	1.5
4		8.6	1.5
5		10.1	1.5
6		8.2	1.5
7		9.5	1.5
8		9.5	1.5
9		9.1	1.5
10		9.0	1.5

MP4

Result	Off	Off
Upper limit		
Standard		
Lower limit		
#	10	10
Average	7.2	1.5
Stdabw	0.8	0
Min.	5.9	1.5
Range	2.4	0
Sigma	0.8	0
3 Sigma	2.4	0

#	Judge	Thick[um]	RefractiveIndex
1		7.7	1.5
2		7.7	1.5
3		5.9	1.5
4		7.9	1.5
5		8.3	1.5
6		7.2	1.5
7		7.0	1.5
8		7.4	1.5
9		6.0	1.5
10		6.6	1.5

MP5

Result	Off	Off
Upper limit		
Standard		
Lower limit		
#	10	10
Average	8.4	1.5
Stdabw	1.5	0
Min.	7.0	1.5
Range	4.6	0
Sigma	1.5	0
3 Sigma	4.5	0

#	Judge	Thick[um]	RefractiveIndex
1		7.8	1.5
2		8.0	1.5
3		9.8	1.5
4		7.1	1.5
5		9.9	1.5
6		7.0	1.5
7		7.6	1.5
8		7.6	1.5
9		7.9	1.5
10		11.6	1.5

MP6

Result	Off	Off
Upper limit		
Standard		
Lower limit		
#	10	10
Average	8.7	1.5
Stdabw	0.6	0
Min.	7.5	1.5
Range	2.0	0
Sigma	0.6	0
3 Sigma	1.8	0

#	Judge	Thick[um]	RefractiveIndex
1		8.6	1.5
2		8.7	1.5
3		7.5	1.5
4		9.2	1.5
5		8.8	1.5
6		8.8	1.5
7		9.2	1.5
8		9.5	1.5
9		8.6	1.5
10		7.9	1.5

MP7

Result	Off	Off
Upper limit		
Standard		
Lower limit		
#	10	10
Average	8.1	1.5
Stdabw	0.5	0
Min.	7.4	1.5
Range	1.4	0
Sigma	0.5	0
3 Sigma	1.5	0

#	Judge	Thick[um]	RefractiveIndex
1		7.6	1.5
2		7.4	1.5
3		7.7	1.5
4		7.9	1.5
5		8.6	1.5
6		8.0	1.5
7		8.8	1.5
8		8.2	1.5
9		7.9	1.5
10		8.7	1.5

MP8

Result	Off	Off
Upper limit		
Standard		
Lower limit		
#	10	10
Average	7.8	1.5
Stdabw	0.7	0
Min.	6.4	1.5
Range	2.9	0
Sigma	0.7	0
3 Sigma	2.2	0

#	Judge	Thick[um]	RefractiveIndex
1		7.5	1.5
2		7.9	1.5
3		8.0	1.5
4		7.1	1.5
5		7.7	1.5
6		7.7	1.5
7		9.3	1.5
8		6.4	1.5
9		8.2	1.5
10		7.8	1.5
